# Symptoms in first-degree relatives of patients with rheumatoid arthritis: evaluation of cross-sectional data from the symptoms in persons at risk of rheumatoid arthritis (SPARRA) questionnaire in the PRe-clinical EValuation of Novel Targets in RA (PREVeNT-RA) Cohort

**DOI:** 10.1186/s13075-021-02593-w

**Published:** 2021-08-11

**Authors:** R. E. Costello, J. H. Humphreys, J. C. Sergeant, M. Haris, F. Stirling, K. Raza, D. van Schaardenburg, Ian N. Bruce

**Affiliations:** 1 Centre for Epidemiology Versus Arthritis, Centre for Musculoskeletal Research, Manchester Academic Health Science Centre, The University of Manchester, Manchester, UK; 2 Kellgren Centre for Rheumatology, Manchester University NHS Foundation Trust, Manchester, UK; 3 Centre for Biostatistics, School of Health Sciences, The University of Manchester, Manchester Academic Health Science Centre, Manchester, UK; 4 NIHR Birmingham Biomedical Research Centre, Birmingham, UK; 5 Research into Inflammatory Arthritis Centre Versus Arthritis and MRC-Versus Arthritis Centre for Musculoskeletal Ageing Research, University of Birmingham, Birmingham, UK; 6 Sandwell and West Birmingham NHS Trust, Birmingham, UK; 7 Amsterdam Rheumatology and immunology Center, location Reade and Amsterdam University Medical Center, Amsterdam, the Netherlands; 8 NIHR Manchester Biomedical Research Centre, Manchester University NHS Foundation Trust, Manchester Academic Health Science Centre, Manchester, UK; 9 Centre for Epidemiology Versus Arthritis, Division of Musculoskeletal and Dermatological Sciences, School of Biological Sciences, Faculty of Biology, Medicine and Health, The University of Manchester, Manchester, M13 9PL UK

**Keywords:** Rheumatoid arthritis, Pre-RA, Arthralgia, Prevention, Epidemiology, Autoantibodies, Risk factors

## Abstract

**Background:**

First-degree relatives (FDRs) of people with rheumatoid arthritis (RA) have a fourfold increased risk of developing RA. The Symptoms in Persons At Risk of Rheumatoid Arthritis (SPARRA) questionnaire was developed to document symptoms in persons at risk of RA. The aims of this study were (1) to describe symptoms in a cohort of FDRs of patients with RA overall and stratified by seropositivity and elevated CRP and (2) to determine if patient characteristics were associated with symptoms suggestive of RA.

**Methods:**

A cross-sectional study of FDRs of patients with RA, in the PREVeNT-RA study, who completed a study questionnaire, provided a blood sample measured for rheumatoid factor, anti-CCP and CRP and completed the SPARRA questionnaire. Moderate/severe symptoms and symmetrical, small and large joint pain were identified and described. Symptoms associated with both seropositivity and elevated CRP were considered suggestive of RA. Logistic regression was used to determine if symptoms suggestive of RA were associated with patient characteristics.

**Results:**

Eight hundred seventy participants provided all data, 43 (5%) were seropositive and 122 (14%) had elevated CRP. The most frequently reported symptoms were sleep disturbances (20.3%) and joint pain (17.9%). Symmetrical and small joint pain were 11.3% and 12.8% higher, respectively, in those who were seropositive and 11.5% and 10.7% higher in those with elevated CRP. In the logistic regression model, seropositivity, older age and feeling depressed were associated with increased odds of small and symmetrical joint pain.

**Conclusions:**

This is the first time the SPARRA questionnaire has been applied in FDRs of patients with RA and has demonstrated that the presence of symmetrical and small joint pain in this group may be useful in identifying people at higher risk of developing RA.

**Supplementary Information:**

The online version contains supplementary material available at 10.1186/s13075-021-02593-w.

## Background

It is known that early treatment of RA improves long-term outcomes and reduces the risk of joint damage [1]. Studies have shown that patients with RA may have circulating autoantibodies 5 or more years prior to developing the disease [2]. Recent studies have also investigated whether treatment prior to a clinical diagnosis of RA can actually prevent disease onset. Results from the PRAIRI (prevention of clinically manifest RA by B cell directed therapy in the earliest phase of disease) study has shown that a single infusion of rituximab in patients who were seropositive (positive for rheumatoid factor (RF) or anti-citrullinated protein antibodies (ACPA)) but without clinical synovitis can delay onset of arthritis, though not prevent it [3]. Further studies are ongoing in patients deemed at high risk of developing RA, usually based on the presence of autoantibodies [4]. Identifying people who are at higher risk of developing RA is not straightforward however, as the aetiology of RA is incompletely understood and involves the accumulation of both genetic and environmental factors [5].

A preclinical phase, where patients experience symptoms prior to the development of overt clinical synovitis, has been described [6]. The EULAR study group for risk factors for RA indicated the importance of identifying symptoms that are associated with subsequent development of RA [6]. Few studies have tried to elucidate this complex of symptoms; however, a qualitative study in 2017 of symptoms in newly diagnosed patients with RA and patients with arthralgia identified themes of joint pain, joint swelling and redness, joint stiffness, weakness and loss of motor control, fatigue, sleeping difficulties and depressive symptoms and pattern of symptoms and onset [7]. Often prediction models predicting RA include symptoms such as swollen and tender joints and morning stiffness [8, 9].

Given the genetic predisposition to RA, [5] first-degree relatives (FDRs) of people with RA are a group at higher risk of developing the disease, with a 2–4 times higher risk compared to the general population [10]. This group may therefore offer an opportunity to study the development of RA in the pre-clinical phase. A study of FDRs of patients with RA in North American native (NAN) population found that FDRs reported more joint symptoms than white controls [11]. However, the prevalence of rheumatic disease in the NAN population is one of the highest in the world [12] thus may not be representative of other groups. Therefore, in this study, we aimed to (1) describe symptoms in a cohort of FDRs of patients with RA overall, then stratified by seropositivity and elevated CRP, and (2) determine any patient characteristics associated with symptoms suggestive of RA.

## Methods

### Study setting

This was a cross-sectional study using data from the PRe-clinical EValuation of Novel Targets in RA (PREVeNT-RA) study, a cohort study of FDRs of patients with established RA in the UK. To be included in the cohort, individuals needed to be an FDR (parent, sibling or half-sibling, offspring) of a proband with a diagnosis of RA (from a rheumatologist) and aged 30 years or over. Individuals were excluded if they had a previous diagnosis of RA or any other inflammatory arthritis.

### Study procedures

Individuals who were eligible for inclusion and consented to take part in the PREVeNT-RA study completed a baseline questionnaire and were provided with a blood collection kit. The baseline questionnaire collected information on demographics, medical history, female reproductive history, lifestyle characteristics, health status measured using the EQ-5D-3L health questionnaire [13] and disability measured using the Health Assessment Questionnaire (HAQ) [14]. The blood collection kit enabled a blood sample to be taken in primary care, or at a local rheumatology clinic, and returned to the study coordinating centre. Individuals who consented to completing further questionnaires were sent the Symptoms in Persons At Risk of Rheumatoid Arthritis (SPARRA) questionnaire (described below) [15] to complete and return to the study coordinating centre. To be included in this analysis, individuals needed to have completed a baseline questionnaire, provided a blood sample and completed the SPARRA questionnaire.

### Blood samples

Blood samples collected prior to SPARRA completion were used to measure rheumatoid factor (RF) (latex test), anti-cyclic citrullinated peptide (anti-CCP) (Viapath CCP – Immunocap) and C-reactive protein (CRP) (Cormay hsCRP assay). Individuals were considered seropositive if RF was ≥ 20 U/ml and/or anti-CCP was ≥ 7 U/ml. Individuals were considered to have high CRP if CRP was ≥ 5 mg/L.

### Cohort characteristics

The following characteristics were determined from the baseline questionnaire: age, gender, smoking status (never smoker, former smoker or current smoker), average alcohol units per week and body mass index (BMI) calculated from self-reported height and weight. Individuals were considered to have depression if they indicated they were moderately or extremely anxious or depressed in the EQ-5D. Education status was categorised by age of leaving education (less than 16 years, 16–17 years, 18–20 years and 21 and over). Socioeconomic status was measured using index of multiple deprivation (IMD) quintile [16]. For females, only the following characteristics were determined: ever being pregnant, ever breastfed a child, ever taken oral contraception and ever taken hormone replacement therapy (HRT).

### SPARRA questionnaire

The SPARRA questionnaire (Additional file 1) collects self-reported information on the duration, severity and impact of joint symptoms: pain, swelling, stiffness, burning sensations, tingling sensations and changes in skin colour; other symptoms: muscle cramps, weakness, fatigue, emotional distress, concentration difficulties and sleep problems; and severity of pain in specific locations in the body [15]. For the purposes of analysis, each symptom was categorised as being severe if severity was reported as either moderate or severe. Patterns of joint pain or swelling indicative of RA were also identified: symmetrical joint pain was identified if moderate or severe pain was indicated in any joint on both sides of the body. Small joint pain was identified if moderate or severe pain was indicated in the fingers, wrist or toes. Large joint pain was identified if moderate or severe pain was indicated in the elbow, shoulder, hip, knee or ankle.

### Analysis

Seropositivity was considered an indicator of ‘higher risk’ of future RA based on previous literature [2] as was elevated CRP given it is a broad marker of inflammation. Cohort characteristics, overall and stratified by seropositivity and elevated CRP, were tabulated. A priori, symmetrical and small joint pain or swelling were considered in keeping with a possible pre-RA phenotype, and a two-sample test of proportions was used to identify the proportion, with 95% confidence interval (CI), of individuals with these features overall and stratified by seropositivity and elevated CRP. The same test was used to identify associations between any other severe symptoms within SPARRA and seropositivity or elevated CRP. Where significant associations were identified, they were considered suggestive of RA.

Subsequently, characteristics associated with the development of RA from previous literature (age, sex, smoking status, alcohol consumption, BMI, education level, IMD quintile, depression, elevated CRP and antibody positivity) [17] were modelled in univariate and multivariate logistic regression models to determine if they were associated with each of the SPARRA items suggestive of RA identified in the first analysis. As there are some female specific characteristics known to be associated with RA development [17], further analyses were conducted in females only with the following additional characteristics included: pregnancy, breastfeeding, taking HRT and taking oral contraceptives. Symptoms not identified as suggestive of RA were modelled in univariate and multivariate (i.e. all symptoms together) logistic regression models to determine if they were associated with the items suggestive of RA.

## Results

**Fig. 1:**
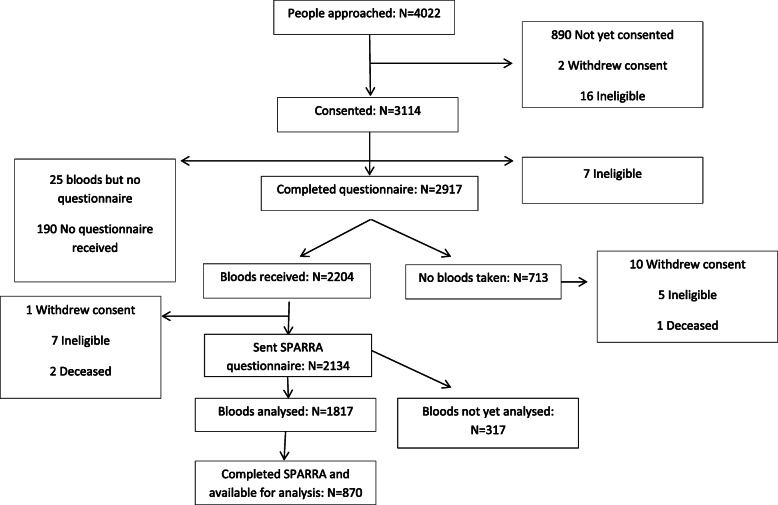
Flowchart of patients included in analysis

As of July 2018, 3114 people had consented to take part in PREVeNT-RA and 2917 had completed the baseline questionnaire. Of those, 713 had not provided a blood sample, 70 had not been sent a SPARRA questionnaire, 317 had not had their blood sample analysed and 947 had not returned the SPARRA questionnaire resulting in 870 people who were eligible for this analysis (Fig. 1). Those eligible for this study were similar in terms of age and gender to those who were not eligible, because either they did not return the SPARRA questionnaire or had returned the SPARRA questionnaire but had not had their blood sample analysed (data not shown).

The mean age of the cohort was 51.8 years (SD 12.1) and 76.6% (*n* = 666) were female. A small proportion were current smokers (6.4%, *n* = 56), and the majority of the cohort were Caucasian (97.8%, *n* = 851). Nearly one fifth reported depression (19.4%, *n* = 169), and the most frequently reported co-morbidity was hypertension (15.4%, *n* = 134). Half of the cohort were in the two least deprived IMD quintiles (IMD quintile 4: 24.4%, *n* = 212, IMD quintile 5: 25.3%, *n* = 220) (Table 1).

**Table 1:** Characteristics of cohort overall and stratified by seropositivity and elevated CRP (*N* = 870)^a^

	Overall	Seropositive			Elevated CRP		
		No	Yes	*P* value	No	Yes	*P* value
*N*	870	827	43		748	122	
Sex = female	666 (76.6)	632 (76.4)	34 (79.1)	0.83	564 (75.4)	102 (83.6)	0.062
Age (mean (SD^b^))	51.78 (12.1)	51.57 (12.1)	55.67 (10.6)	0.03	51.72 (12.1)	52.12 (11.9)	0.741
BMI^b^ category				0.727			< 0.001
Underweight	14 ( 1.7)	14 ( 1.7)	0 ( 0.0)		13 ( 1.8)	1 ( 0.9)	
Normal weight	374 (44.2)	357 (44.4)	17 (40.5)		349 (47.9)	25 (21.4)	
Overweight	293 (34.6)	276 (34.3)	17 (40.5)		258 (35.4)	35 (29.9)	
Obese	165 (19.5)	157 (19.5)	8 (19.0)		109 (15.0)	56 (47.9)	
Age left education				0.836			0.035
Less than 16 years	51 ( 6.0)	49 ( 6.0)	2 ( 4.8)		43 ( 5.8)	8 ( 6.7)	
16–17 years	333 (38.9)	319 (39.1)	14 (33.3)		278 (37.7)	55 (46.2)	
18–20 years	209 (24.4)	198 (24.3)	11 (26.2)		176 (23.8)	33 (27.7)	
21+ years	264 (30.8)	249 (30.6)	15 (35.7)		241 (32.7)	23 (19.3)	
Smoking status				0.729			0.08
Never smoker	498 (57.3)	471 (57.0)	27 (62.8)		439 (58.8)	59 (48.4)	
Former smoker	315 (36.2)	301 (36.4)	14 (32.6)		260 (34.8)	55 (45.1)	
Current smoker	56 ( 6.4)	54 ( 6.5)	2 ( 4.7)		48 ( 6.4)	8 ( 6.6)	
Ethnicity				0.001			0.094
White	851 (97.9)	811 (98.2)	40 (93.0)		734 (98.3)	117 (95.9)	
Mixed	4 ( 0.5)	4 ( 0.5)	0 ( 0.0)		4 ( 0.5)	0 ( 0.0)	
Asian	8 ( 0.9)	5 ( 0.6)	3 ( 7.0)		6 ( 0.8)	2 ( 1.6)	
Black	4 ( 0.5)	4 ( 0.5)	0 ( 0.0)		2 ( 0.3)	2 ( 1.6)	
Chinese	2 ( 0.2)	2 ( 0.2)	0 ( 0.0)		1 ( 0.1)	1 ( 0.8)	
Average units of alcohol per week (mean (SD^b^))	7.44 (8.83)	7.40 (8.76)	8.21 (10.20)	0.559	7.53 (8.94)	6.90 (8.14)	0.472
Diabetes mellitus	28 ( 3.2)	23 ( 2.8)	5 (11.9)	0.005	20 ( 2.7)	8 ( 6.6)	0.048
Psoriasis	38 ( 4.4)	37 ( 4.5)	1 ( 2.4)	0.789	30 ( 4.0)	8 ( 6.6)	0.298
Hypertension	134 (15.5)	126 (15.3)	8 (18.6)	0.714	104 (14.0)	30 (24.8)	0.003
Depression	169 (19.7)	164 (20.1)	5 (11.6)	0.245	138 (18.6)	31 (26.3)	0.068
IMD^b^ quintile				0.587			0.749
1 (most deprived)	64 ( 7.9)	61 ( 7.9)	3 ( 7.5)		56 ( 8.0)	8 ( 6.8)	
2	133 (16.3)	126 (16.3)	7 (17.5)		109 (15.6)	24 (20.5)	
3	186 (22.8)	181 (23.4)	5 (12.5)		160 (22.9)	26 (22.2)	
4	212 (26.0)	199 (25.7)	13 (32.5)		182 (26.1)	30 (25.6)	
5 (least deprived)	220 (27.0)	208 (26.8)	12 (30.0)		191 (27.4)	29 (24.8)	

^a^Data are shown as number (percentage) unless otherwise indicated. ^b^SD, standard deviation; BMI, body mass index; IMD, Index of Multiple Deprivation

In this cohort, 5% (*n* = 43) were seropositive, primarily RF positive (Additional file 2, Table 1) and 14% (*n* = 122) had elevated CRP. Those who were seropositive were older, had a higher proportion with Asian ethnicity and with diabetes. Those with elevated CRP had a higher BMI, fewer years of education, were more likely to have smoked and more likely to have hypertension and diabetes (Table 1).

### Symptoms

**Fig. 2:**
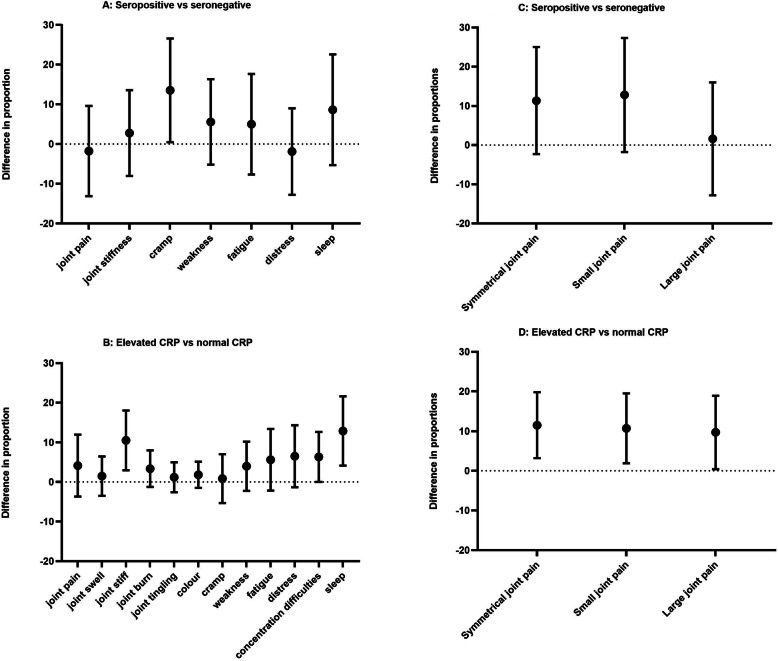
Interval plots of symptoms and patterns of joint symptoms stratified by seropositivity and elevated CRP

The most frequently reported symptoms were sleep disturbances (20.3%), joint pain (17.9%), fatigue (16.7%) and distress (16.1%). When stratified by seropositivity, the proportion reporting muscle cramps was significantly higher in those who were seropositive (seronegative: *n* = 84, 10.3% vs seropositive: *n* = 10, 23.8%) (Table 2 and Fig. 2A). When stratified by elevated CRP, those with elevated CRP had significantly more joint stiffness (normal CRP: *n* = 44, 6% vs elevated CRP: *n* = 25, 20.7%), concentration difficulties (normal CRP: *n* = 51, 6.9% vs elevated CRP *n* = 16, 13.2%) and sleep disturbances (normal CRP: *n* = 137, 18.5% vs elevated CRP: *n* = 38, 31.4%) (Table 2 and Fig. 2B).

**Table 2:** Symptoms and pattern of joint pain, overall and stratified by seropositivity and elevated CRP

	Overall	Seronegative	Seropositive	Difference in proportions^a, b^ (95% CI)	Normal CRP	Elevated CRP	Difference in proportions^a^ (95% CI)
*N*	870	827	43		748	122	
Symptoms							
Joint pain	154 (17.9)	147 (18.0)	7 (16.3)	− 1.76 (− 13.1 to 9.59)	128 (17.4)	26 (21.5)	4.12 (− 3.69 to 11.93)
Joint swelling	53 ( 6.2)	52 ( 6.4)	1 ( 2.4)		44 ( 6.0)	9 ( 7.4)	1.48 (− 3.5 to 6.45)
Joint stiffness	100 (11.7)	94 (11.5)	6 (14.3)	2.77 (− 8.04 to 13.57)	75 (10.2)	25 (20.7)	10.48 (2.95 to 18.02)
Joint burning	32 ( 3.7)	31 ( 3.8)	1 ( 2.4)		24 ( 3.3)	8 ( 6.6)	3.35 (− 1.26 to 7.96)
Joint tingling	27 ( 3.2)	27 ( 3.3)	0 ( 0.0)		22 ( 3.0)	5 ( 4.2)	1.18 (− 2.6 to 4.96)
Colour change	15 ( 1.8)	15 ( 1.8)	0 ( 0.0)		11 ( 1.5)	4 ( 3.3)	1.81 (− 1.49 to 5.12)
Cramp	94 (10.9)	84 (10.3)	10 (23.8)	13.53 (0.48 to 26.58)	80 (10.8)	14 (11.7)	0.84 (− 5.32 to 7.01)
Weakness	77 ( 9.0)	71 ( 8.7)	6 (14.3)	5.57 (− 5.18 to 16.33)	62 ( 8.4)	15 (12.4)	3.97 (− 2.23 to 10.18)
Fatigue	143 (16.7)	134 (16.4)	9 (21.4)	4.99 (− 7.68 to 17.65)	117 (15.9)	26 (21.5)	5.59 (− 2.19 to 13.37)
Distress	138 (16.1)	132 (16.2)	6 (14.3)	− 1.89 (− 12.77 to 8.99)	112 (15.2)	26 (21.7)	6.49 (− 1.32 to 14.3)
Concentration difficulties	67 ( 7.8)	66 ( 8.1)	1 ( 2.4)		51 ( 6.9)	16 (13.2)	6.31 (0.01 to 12.62)
Sleep disturbances	175 (20.3)	163 (19.9)	12 (28.6)	8.64 (− 5.29 to 22.58)	137 (18.5)	38 (31.4)	12.87 (4.13 to 21.6)
Pattern of joint pain							
Symmetrical joint pain	149 (17.1)	137 (16.6)	12 (27.9)	11.34 (− 2.3 to 24.99)	116 (15.5)	33 (27.0)	11.54 (3.24 to 19.84)
Small joint pain	198 (22.8)	183 (22.1)	15 (34.9)	12.76 (− 1.77 to 27.28)	159 (21.3)	39 (32.0)	10.71 (1.93 to 19.49)
Large joint pain	270 (31.0)	256 (31.0)	14 (32.6)	1.6 (− 12.75 to 15.96)	222 (29.7)	48 (39.3)	9.67 (0.4 to 18.93)

^a^Difference in proportions is considered significantly different from zero if the 95% CI does not include zero

^b^Difference in proportions not able to be calculated if the number in one strata is less than 5 as the CI cannot be (reliably) calculated

### Patterns of joint symptoms

The most frequently identified pattern of joint pain was large joint pain (31%, *n* = 270). One fifth of the cohort reported small joint pain (22.8%, *n* = 198) and 17.1% (*n* = 149) reported symmetrical joint pain. When stratified by seropositivity, a higher proportion of those who were seropositive had small joint pain (seronegative: *n* = 183, 22.1% vs seropositive: *n* = 15, 34.9%) and symmetrical joint pain (seronegative: *n* = 137, 16.6% vs seropositive: *n* = 12, 27.9%)), though these differences were not statistically significant (Table 2 and Fig. 2C). When stratified by elevated CRP, a significantly higher proportion of those with elevated CRP had symmetrical (normal CRP: *n* = 116, 15.5% vs elevated CRP: *n* = 33, 27.0%), small joint (normal CRP: *n* = 159, 21.3% vs elevated CRP: *n* = 39, 32.0%) and large joint pain (normal CRP: *n* = 222, 29.7% vs elevated CRP: *n* = 48, 39.3%) (Table 2 and Fig. 2D). Only a small number of participants reported joint swelling (*n* = 52); almost all were seronegative (*n* = 51) and had normal CRP (*n* = 44) (Table 2). Therefore, further analysis of joint swelling including stratifying by size and symmetry of joints involved was not possible.

### Characteristics associated with small and symmetrical joint pain

From first principles, having both symmetrical and small joint pain was considered suggestive of RA and therefore was incorporated as an alternative outcome measure to seropositivity. There were 116/870 (13.3%) people who had both symmetrical and small joint pain. For characteristics, the multivariate logistic regression model indicated that higher age (OR 1.04 (95% CI 1.02, 1.07)), feeling depressed (OR 2.77 (95% CI 1.65, 4.65)) and being antibody positive (OR 2.55 (95% CI 1.09, 5.97)) were associated with increased odds of having both symmetrical and small joint pain. Leaving education age 18–20 years, compared to leaving education age 16–17 years, was associated with reduced odds of symmetrical and small joint pain (OR 0.51 (95% CI 0.27, 0.97) (Table 3). In the model with females only, higher age (OR 1.03 (95% CI 1.01, 1.06)) and depression (OR 2.92 (95% CI 1.58, 5.39)) remained associated with increased odds of symmetrical and small joint pain. In addition, ever using HRT was associated with increased odds of symmetrical and small joint pain (OR 2.21 (95% CI 1.15, 4.23)) (Additional file 2, Table 2).

**Table 3:** Characteristics and associations with symmetrical and small joint pain (*N* = 764)^a^

Characteristic		Number (%) without symmetrical and small joint pain, unless otherwise specified	Number (%) with small and symmetrical joint pain, unless otherwise specified	Univariate logistic regression odds ratio (95% CI)^c^	Multivariate logistic regression odds ratio (95% confidence interval)^b^
Age (continuous, mean (SD^c^))		50.9 (11.9)	57.4 (12.0)	1.05 (1.03, 1.06)	1.04 (1.02, 1.07)
Sex	Male	156 (87.2)	23 (12.9)	Reference	Reference
	Female	514 (87.9)	71 (12.1)	0.94 (0.57, 1.55)	1.18 (0.67, 2.06)
Smoking status	Never smoker	401 (89.7)	46 (10.3)	Reference	Reference
	Former smoker	235 (85.5)	40 (14.6)	1.48 (0.94, 2.33)	1.28 (0.78, 2.11)
	Current smoker	34 (81.0)	8 (19.1)	2.05 (0.90, 4.70)	1.55 (0.61, 3.92)
Average units of alcohol consumed per week (continuous) (mean (SD^c^))		7.5 (8.8)	7.0 (8.9)	1.00 (0.97, 1.02)	0.99 (0.97, 1.02)
BMI^c^ category	Underweight	13 (100)	0	Null	Null
	Normal weight	309 (91.7)	28 (8.3)	Reference	Reference
	Overweight	220 (84.0)	42 (16.0)	2.11 (1.27, 3.50)	1.57 (0.91, 2.71)
	Obese	128 (84.2)	24 (15.8)	2.07 (1.16, 3.71)	1.63 (0.84, 3.17)
Educational age left education	Less than 16 years	35 (74.5)	12 (25.5)	1.79 (0.87, 3.71)	0.84 (0.37, 1.91)
	16–17 years	246 (84.0)	47 (16.0)	Reference	Reference
	18–20 years	176 (92.2)	15 (7.9)	0.45 (0.24, 0.82)	0.51 (0.27, 0.97)
	21+ years	213 (91.4)	20 (8.6)	0.49 (0.28, 0.86)	0.63 (0.35, 1.14)
IMD^c^ quintile	1	48 (80.0)	12 (20.0)	Reference	Reference
	2	109 (89.3)	13 (10.7)	0.48 (0.20, 1.12)	0.51 (0.2, 1.31)
	3	158 (89.8)	18 (10.2)	0.46 (0.21, 1.01)	0.47 (0.2, 1.13)
	4	171 (86.4)	27 (13.6)	0.63 (0.30, 1.34)	0.65 (0.28, 1.53)
	5	184 (88.5)	24 (11.5)	0.52 (0.24, 1.12)	0.51 (0.21, 1.22)
Depression	No	564 (90.4)	60 (9.6)	Reference	Reference
	Yes	106 (75.7)	34 (24.3)	3.02 (1.89, 4.82)	2.77 (1.65, 4.65)
CRP^c^	Normal	587 (88.7)	75 (11.3)	Reference	Reference
	Elevated	83 (81.4)	19 (20.2)	1.79 (1.03, 3.12)	1.19 (0.63, 2.25)
Antibody status	Seronegative	642 (88.3)	85 (11.7)	Reference	Reference
	Seropositive	28 (75.7)	9 (24.3)	2.43 (1.11, 5.32)	2.55 (1.09, 5.97)

^a^Included only complete cases. ^b^ Odds ratio is considered significantly different from one if the 95% CI does not include one. ^c^SD, standard deviation; BMI, body mass index; IMD, Index of Multiple Deprivation, CRP: C-reactive protein

### Symptoms associated with small and symmetrical joint pain

Those with small and symmetrical joint pain reported more symptoms overall. The multivariate logistic regression model indicated that joint pain, joint stiffness, joint burning, joint tingling and fatigue were all associated with increased odds of symmetrical and small joint pain (Table 4). Similar results were seen in the female only model (Additional file 2, Table 3).

**Table 4:** Symptoms and associations with symmetrical and small joint pain (*N* = 829)

Symptom present	Number without symmetrical and small joint pain (%)	Number with small and symmetrical joint pain (%)	Univariate logistic regression odds ratio (95% CI)^$^	Multivariate^a^ logistic regression odds ratio (95% confidence interval)^b^
Joint pain	51 (7.5)	53 (36.8)	7.2 (4.7, 11.3)	2.16 (1.15, 4.09)
Joint swell	84 (10.8)	20 (41.7)	5.9 (3.2, 11.0)	1.03 (0.40, 2.64)
Joint stiff	59 (8.0)	45 (48.4)	10.8 (6.6, 17.5)	3.00 (1.50, 6.00)
Joint burn	88 (11.0)	16 (57.1)	10.8 (4.9, 23.6)	2.91 (1.03, 8.24)
Joint tingling	85 (10.6)	19 (73.1)	22.9 (9.4, 56.1)	7.32 (2.49, 21.49)
Colour	100 (12.3)	4 (28.6)	2.9 (0.9, 9.3)	0.52 (0.10, 2.84)
Cramp	78 (10.6)	26 (28.9)	3.4 (2.1, 5.7)	1.47 (0.75, 2.88)
Weakness	73 (9.7)	31 (42.5)	6.9 (4.1, 11.7)	1.28 (0.61, 2.70)
Fatigue	56 (8.1)	48 (34.5)	6.0 (3.8, 9.3)	2.03 (1.06, 3.89)
Distress	69 (9.9)	35 (6.5)	3.3 (2.1, 5.2)	0.89 (0.44, 1.79)
Concentration difficulties	80 (10.4)	24 (39.3)	5.6 (3.2, 9.8)	0.88 (0.36, 2.15)
Sleep disturbances	58 (8.8)	46 (27.2)	3.9 (2.5, 6.0)	1.31 (0.71, 2.42)

^a^All symptoms modelled together, with no other adjustment. ^b^ Odds ratio is considered significantly different from one if the 95% CI does not include one

## Discussion

This is the first time that symptoms have been captured in a cohort of FDRs of patients with RA, using a questionnaire that that captures data related to the full spectrum of symptoms associated with the early stages of RA. We found that symmetrical and small joint pain was associated with antibody positivity and higher levels of inflammation in the blood. The study also identified associations between increasing age, depression and seropositivity and odds of having both symmetrical and small joint pain; whereas education after age 16 was associated with reduced odds of having both symmetrical and small joint pain. Other reported symptoms did not show a clear pattern helpful for identifying patients at risk of RA in a cross-sectional setting; this may be due to the broad nature of the symptoms. Symmetrical and small joint pain have also been associated with RA in previous studies [17], highlighting that the sections of the SPARRA questionnaire where symmetrical and small joint pain can be identified may be useful for identifying people at high risk of developing RA.

It was of interest that depression was strongly associated with symmetrical and small joint pain. Depression has been found to be highly prevalent in patients with RA [18]. Depression and RA have also been shown to have a bidirectional association, with the presence of pre-existing depression increasing the likelihood of developing RA, and the presence of RA increasing the likelihood of developing depression [19]. Further, in a study of patients with seropositive arthralgia, depressive mood was found to be associated with musculoskeletal symptoms but not arthritis development [20], though recent studies have found depression to be associated with subsequent RA [21, 22]. In our study, depression was highly associated with having small and symmetrical joint pain and also large joint pain (results not shown). However, depression in our study was measured relatively crudely with a single question in the EQ-5D questionnaire, and therefore, this relationship warrants further investigation in other pre-RA cohorts. Our finding that low educational attainment was associated with symmetrical and small joint pain is also in keeping with previous inflammatory arthritis literature; in the EPIC to NOAR study, Lahiri et al. identified that rates of inflammatory polyarthritis were lower in those with degree level education [16].

A surprising finding was that the proportion of current and former smokers was lower in those who were seropositive compared to those who were seronegative. This is most likely to be due to the small number of people who were seropositive (only 5% of this cohort), though the proportion who were seropositive was similar in a Dutch FDR cohort with similar patient characteristics, where 6.6% were seropositive [23]. The subset of the PREVeNT-RA cohort used for this study had few comorbidities and may have had healthier lifestyles than the broader UK population. For example, the proportion of the cohort who were current smokers was low, and only 6.4% were current smokers, which is lower than current UK wide estimates of 15% [24]. Similarly, only 3.2% of participants self-reported diabetes, whereas the 2016 estimated prevalence in England was 8.6% [25]. Overall 52.7% had a BMI that was overweight or obese which is lower than 64% estimated to be overweight or obese in the UK in 2017 [26]. However, a high proportion (19.4%) indicated feeling moderately or severely anxious or depressed, slightly greater than the overall UK estimates of 15.7% [27]. This means the study population may not be fully representative of the general FDR population, perhaps due to non-responder bias, as to be included in this study participants needed to have completed and returned the SPARRA questionnaire.

Only a few studies to date have sought to describe symptoms in unaffected FDRs of patients with RA, mainly reporting joint symptoms. Our findings were similar those of a study of unaffected FDRs in the USA (the SERA cohort) where 23% reported inflammatory joint signs, defined as tender or swollen RA-specific joints (hands, wrists, feet and elbow) [28] which is similar to the proportion reporting small joint pain in this study (22.8%). However, in a different study of NAN FDRs, joint symptoms were more frequently reported compared to this study, with 55% reporting pain in the hands compared to 18% reporting pain in any joint in this study [11]; this may be due to differences between the populations used as NANs have higher prevalence of RA [12]. Musculoskeletal symptoms have also been described in a non-FDR population, where symptoms in consulters to UK primary care with a musculoskeletal (MSK) condition were compared to symptoms in a matched population who consulted for a non-MSK condition. It was found 42% MSK consulters reported symmetrical joint pain, so too did 37% of the matched sample [29]. For both groups, this was higher than in our study where 17% reported symmetrical joint pain. However, the patients in that study were older than in our study and likely to be more unwell as they were consulting at primary care. Studies of patients who were seropositive, but not specifically FDRs, found similar results to our study. Rakieh et al found that found tenderness in small joints to be associated with progression to inflammatory arthritis in patients who were anti-CCP positive [9]. Another small study included 10 people who were ACPA-positive or IgM RF-positive and had symmetrical small joint arthralgia; of those, 6 went onto to develop arthritis [30]. This suggests symmetrical and small joint pain may be important to identify in at-risk populations and that questionnaires such as this will have utility on screening such groups.

This was a large prospective study of unaffected FDRs of patients with RA in the PREVeNT-RA cohort, and the use of the SPARRA questionnaire allowed the collection of detailed symptom data. However, there were some limitations. It was a cross-sectional study; thus, we cannot infer any time dependent or causal relationships. Although the PREVeNT-RA study has been running for 7 years, there are currently too few cases of RA to analyse and validate the SPARRA questionnaire against the outcome of a clinical RA diagnosis. We therefore used seropositivity as proxy measures of this outcome, indicating those at ‘higher risk’ of future RA. We acknowledge, however, that not all patients with RA are seropositive nor all seropositive individuals develop RA [5], particularly those who are only RF positive [30]. Our study was also not able to recall these patients for more detailed clinical assessment, such as joint examination; therefore, there may have been undiagnosed RA or other inflammatory conditions. This could mean some characteristics identified as putting a person at high risk of RA, could be symptoms of RA that has already developed, or conversely could be due to another condition. Enhancing this questionnaire with additional clinical and imaging assessments may identify RA at a very early stage for interventions, and such future studies are planned. Finally, our response rate to the questionnaire was low, albeit within the range expected for surveys [31]. The SPARRA questionnaire is long, and some subjects fed back some difficulties in understanding all the questions. Our work however may point the way to how the questionnaire could be refined and perhaps shortened without any loss of its discriminatory power.

## Conclusions

This study of FDRs of patients with RA found that symmetrical and small joint pain was associated with antibody positivity and higher levels of inflammation in the blood, which may be an indicator of people who may be at higher risk of developing RA.

## Supplementary Information

**Additional file 1:.** Symptoms in Persons At Risk of Rheumatoid Arthritis (SPARRA) Questionnaire

**Additional file 2: **Table 1 Specific details of seropositivty. Table 2 Characteristics and associations with symmetrical and small joint pain in females only. Table 3: Symptoms and associations with symmetrical and small joint pain in females only (*N*=631)

## Data Availability

No data are available as the PREVeNT-RA study is still ongoing. **Declarations**
