# Correction to: Symptoms in first-degree relatives of patients with rheumatoid arthritis: evaluation of cross-sectional data from the symptoms in persons at risk of rheumatoid arthritis (SPARRA) questionnaire in the Preclinical EValuation of Novel Targets in RA (PREVeNT-RA) Cohort

**DOI:** 10.1186/s13075-021-02645-1

**Published:** 2021-10-19

**Authors:** R. E. Costello, J. H. Humphreys, J. C. Sergeant, M. Haris, F. Stirling, K. Raza, D. van Schaardenburg, Ian N. Bruce

**Affiliations:** 1 Centre for Epidemiology Versus Arthritis, Centre for Musculoskeletal Research, Manchester Academic Health Science Centre, The University of Manchester, Manchester, UK; 2 Kellgren Centre for Rheumatology, Manchester University NHS Foundation Trust, Manchester, UK; 3 Centre for Biostatistics, School of Health Sciences, The University of Manchester, Manchester Academic Health Science Centre, Manchester, UK; 4 NIHR Birmingham Biomedical Research Centre, Birmingham, UK; 5 Research into Inflammatory Arthritis Centre Versus Arthritis and MRC-Versus Arthritis Centre for Musculoskeletal Ageing Research, University of Birmingham, Birmingham, UK; 6 Sandwell and West Birmingham NHS Trust, Birmingham, UK; 7 Amsterdam Rheumatology and immunology Center, Location Reade and Amsterdam University Medical Center, Amsterdam, the Netherlands; 8 NIHR Manchester Biomedical Research Centre, Manchester University NHS Foundation Trust, Manchester Academic Health Science Centre, Manchester, UK; 9 Centre for Epidemiology Versus Arthritis, Division of Musculoskeletal and Dermatological Sciences, School of Biological Sciences, Faculty of Biology, Medicine and Health, The University of Manchester, Manchester, M13 9PL UK

**Correction to: Arthritis Res Ther 23, 210 (2021)**

**https://doi.org/10.1186/s13075-021-02593-w**

Following publication of the original article [1], the authors have requested to update the Acknowledgement section and added the following statements:

We would like to thank the PREVeNT-RA participants and principal investigators as follows: Dr Michael Batley - Maidstone and Tunbridge Wells NHS Trust, Dr Marwan Bukhari - Royal Lancaster Infirmary, Lancaster, Dr James Burns - Antrim Area Hospital, Northern Ireland, Dr Lucy Coates - Tameside General Hospital, Manchester, Dr Andrew Cope - Guy’s Hospital, London, Dr Emily Deeney - Queen Elizabeth Hospital, Gateshead, Dr Karen Douglas - Russells Hall Hospital, Dudley, Dr Paul Emery - Leeds Teaching Hospital, Dr Andrew Filer - Birmingham Queen Elizabeth hospital, Dr Charlotte Filer - Stepping Hill Hospital, Stockport, Dr James Galloway - Kings College hospital, London, Dr Phillip Gardiner - Altnagelvin Hospital (Western Health and Social Care Trust), Northern Ireland, Dr John Isaacs - Freeman Hospital, Newcastle, Dr Rachel Jeffrey - Northampton General Hospital, Dr Sophia Khan - Heart of England NHS foundation Trust, Solihull, Dr Suzanne Lane - Ipswich Hospital, Suffolk, Dr Sara Littlejohns - North Devon District Hospital, Barnstable, Dr Kirsten Mackay - Torbay Hospital, Torquay, Dr Nicola Maiden - Craigavon Area Hospital, Portadown, Dr Tarnya Marshall - Norfolk and Norwich University Hospital, Norfolk, Dr Sophia Naz - Fairfield General Hospital, Bury, Dr Sophia Naz - North Manchester General Hospital, Manchester, Dr Terence O'Neill - Salford Royal Hospital, Salford, Dr Ira Pande - Queens Medical Centre, Nottingham, Dr Yusef Patel - Hull Royal Infirmary, Hull, Dr Anandita Paul - Bolton NHS Foundation Trust, Dr John Pauling - Royal National Mineral Hospital for Rheumatic Diseases, Bath, Dr Adrian Peall - The County Hospital, Hereford, Dr Suzannah Pegler - Great Western Hospital, Swindon, Dr Helen Prady - Warrington and Halton Teaching Hospitals NHS Foundation Trust, Warrington, Dr Karim Raza - Birmingham City Hospital, Birmingham, Dr Lindsay Robertson - Derriford Hospital, Plymouth, Dr Richard Smith - Salisbury District Hospital, Wiltshire, Dr Peter Taylor - Oxford University Hospitals NHS Foundation Trust, Oxford, Dr Julia Taylor - Poole Hospital, Dorset, Jessica Thrush - Worcestershire Acute Hospitals NHS Trust, Redditch Dr Lisa Trembath - Royal Cornwall Hospital, Truro, Professor David Walsh - Sherwood Forest Hospitals NHS Foundation Trust, Nottinghamshire, Dr Pippa Watson - Manchester University NHS Foundation Trust, Manchester, Professor Simon Bowman - Milton Keynes University Hospital NHS Foundation Trust, Milton Keynes, Helena Cox - Burton Hospitals (Queens Hospital) & Royal Derby Hospital, Dr Emily Deeney - Gateshead Health NHS Foundation Trust, Gateshead, Dr Sam Hider - Haywood Hospital, Stoke, Dr Cath Lawson - Harrogate District Hospital, Harrogate, Dr Paul McCabe - Trafford General Hospital, Manchester, Dr Constantino Pitzalis - Mile End Hospital, London, Dr Lee Suan - The Royal Blackburn Hospital, Blackburn, Dr Louise Warburton - Shropshire Community Health NHS Trust, Shrewsbury.

The original article [1] has been updated.

## References

[1] Costello RE, Humphreys JH, Sergeant JC (2021). Symptoms in first-degree relatives of patients with rheumatoid arthritis: evaluation of cross-sectional data from the symptoms in persons at risk of rheumatoid arthritis (SPARRA) questionnaire in the PRe-clinical EValuation of Novel Targets in RA (PREVeNT-RA) Cohort. Arthritis Res Ther.

