# Supplementary material for: Symptoms in first-degree relatives of patients with rheumatoid arthritis: evaluation of cross-sectional data from the symptoms in persons at risk of rheumatoid arthritis (SPARRA) questionnaire in the PRe-clinical EValuation of Novel Targets in RA (PREVeNT-RA) Cohort
Source: Arthritis Res Ther. 2021 Aug 11;23:210. doi: 10.1186/s13075-021-02593-w (PMC8356426; doi:10.1186/s13075-021-02593-w)
Supplement: Supplementary file 1 — Additional file 1:. Symptoms in Persons At Risk of Rheumatoid Arthritis (SPARRA) Questionnaire [file 13075_2021_2593_MOESM1_ESM.pdf]

Symptoms in Persons At Risk of Rheumatoid Arthritis (SPARRA) Questionnaire

You may or may not have experienced one or more of the following symptoms in relation to your current episode of joint problems. Please indicate whether you have had the listed symptoms, and when they first appeared. For example, if you have had joint pain for the last 3 months, and swelling for the last 4 years but never had stiffness of your joints, you fill in:

Example of possible symptom	Experienced (past or present)*	If yes, how long ago did it first appear?
Joint pain	Past /present / not	3 months ~~/ years~~
Swelling of the joints	Past /present / not	4 ~~months /~~ years
Stiffness of the joints	Past / present/not	~~… months / years~~

In the bottom two rows, there is space to add any other symptoms you may have noticed.

Possible symptom	Experienced (past or present)*	If yes, how long ago did it first appear?*
Joint pain	Past / present / not	… months / years
Swelling of the joints	Past / present / not	… months / years
Stiffness of the joints	Past / present / not	… months / years
Burning sensations in the joints	Past / present / not	… months / years
Tingling sensations in the joints	Past / present / not	… months / years
Numbness in the joints	Past / present / not	… months / years
Changes in skin colour over any joints	Past / present / not	… months / years
Muscle cramps	Past / present / not	… months / years
Weakness or loss of strength	Past / present / not	… months / years
Fatigue	Past / present / not	… months / years
Emotional distress<br>(e.g. sadness, worry, upset)	Past / present / not	… months / years
Concentration difficulties	Past / present / not	… months / years
Sleep problems	Past / present / not	… months / years
Other symptom, namely:<br>______________________________	Past / present	… months / years
Other symptom, namely:<br>______________________________	Past/present	… months / years

If you have had any other symptoms **before this episode of joint problems** that you think may be relevant, you can describe them here:

____

____

The answers to the following questions will help us to understand more about the type of symptoms you have experienced over the **past month.** Do not think too long about the questions; the first answer that comes to mind is often the best.
Please read each question and circle the one option which best answers the question for you, for example:

**Example question**				
**a) Over the past month how many days of the month have you had X in your joints?**	0 days (continue to next question)	1 to 5 days	6 to 15 days	16 to 30 days

**Q1: Joint pain**				
**1a) Over the past month how many days of the month have you had pain in your joints?**	0 days (continue to question 2)	1 to 5 days	6 to 15 days	16 to 30 days
**1b) Over the past month how much joint pain have you had?**	None	Mild	Moderate	Severe
**1c) What impact has this joint pain had on your ability to carry out daily activities (e.g. work, household chores, childcare, social activities)?**	No impact	A small impact	A moderate impact	A large impact
**1d) Which of the following descriptions is most like your joint pain?**	Burning pain	Sharp or stabbing pain	Aching pain	Other type of pain. Please describe: ______________ ______________
**1e) Does your joint pain move from joint to joint?**	No	from arms to legs	from legs to arms	from one side to the other

**Q2: Joint swelling**				
**2a) Over the past month how many days of the month have you had swelling in your joints?**	0 days (continue to question 3)	1 to 5 days	6 to 15 days	16 to 30 days
**2b) Over the past month how much joint swelling have you had?**	None	Mild	Moderate	Severe
**2c) What impact has joint swelling had on your ability to carry out daily activities (e.g. work, household chores, childcare, social activities)?**	No impact	A small impact	A moderate impact	A large impact
**2d) Where did you feel the joint swelling? (circle all that apply)**	Hand: One Both	Arm: One Both	Foot: One Both	Leg: One Both

**Q3: Joint stiffness**				
**3a) Over the past month how many days of the month have you had stiffness in your joints?**	0 days (continue to question 4)	1 to 5 days	6 to 15 days	16 to 30 days
**3b) Over the past month, how much joint stiffness have you had?**	None	Mild	Moderate	Severe
**3c) If you have had joint stiffness when you wake up in the morning how long does it last?**	I don't have morning stiffness	For less than an hour: … minutes**	For 1 to 2 hours	All morning
**3d) What impact has joint stiffness had on your ability to carry out daily activities (e.g. work, household chores, childcare, social activities)?**	No impact	A small impact	A moderate impact	A large impact
**3e) Where did you feel the joint stiffness? (circle all that apply)**	Hand: One Both	Arm: One Both	Foot: One Both	Leg: One Both

** please fill in how many minutes on average

**Q4: Burning sensations in joints**				
**4a) Over the past month how many days of the month have you had a feeling of burning in your joints?**	0 days (continue to question 5)	1 to 5 days	6 to 15 days	16 to 30 days
**4b) Over the past month, how much feeling of burning in your joints have you had?**	Not	Mild	Moderate	Severe
**4c) What impact has this feeling of burning in your joints had on your ability to carry out daily activities (e.g. work, household chores, childcare, social activities)?**	No impact	A small impact	A moderate impact	A large impact
**4d) Where did you** experience this feeling of burning in your joints **? (circle all that apply)**	Hand:<br>One<br>Both	Arm:<br>One<br>Both	Foot:<br>One<br>Both	Leg:<br>One<br>Both

**Q5: Tingling sensations in joints**				
**5a) Over the past month how many days of the month have you had pins and needles or tingling sensations?**	0 days (continue to question 6)	1 to 5 days	6 to 15 days	16 to 30 days
**5b) Over the past month how much tingling have you had?**	None	Mild	Moderate	Severe
**5c) What impact has this tingling had on your ability to carry out daily activities (e.g. work, household chores, childcare, social activities)?**	No impact	A small impact	A moderate impact	A large impact
**5d) Where did you feel the tingling? (circle all that apply)**	Hand:<br>One<br>Both	Arm:<br>One<br>Both	Foot:<br>One<br>Both	Leg:<br>One<br>Both

**Q6: Numbness**				
**6a) Over the past month how many days of the month have you had numbness?**	0 days (continue to question 7)	1 to 5 days	6 to 15 days	16 to 30 days
**6b) Over the past month, how much numbness have you had?**	None	Mild	Moderate	Severe
**6c) What impact has this numbness had on your ability to carry out activities such as e.g. work, household chores, childcare, social activities?**	No impact	A small impact	A moderate impact	severe impact
**6d) Where did you feel the numbness? (circle all that apply)**	Hand: One Both	Arm: One Both	Foot: One Both	Leg: One Both

**Q7: Changes in skin colour over joints (may be skin looking unusually red, blue, brown, etc)**				
**7a) Over the past month how many days of the month have you had skin discolouration over any joints?**	0 days (continue to question 8)	1 to 5 days	6 to 15 days	16 to 30 days
**7b) Over the past month, how much skin discolouration have you had?**	None	Mild	Moderate	Severe
**7c) What impact has skin discolouration had on your ability to carry out day to day activities?**	No impact	A small impact	A moderate impact	A large impact
**7d) Where did you experience skin discolouration? (circle all that apply)**	Hand: One Both	Arm: One Both	Foot: One Both	Leg: One Both

**Q8: Muscle cramps**				
**8a) Over the past month how many days of the month have you had muscle cramps?**	0 days (continue to question 9)	1 to 5 days	6 to 15 days	16 to 30 days
**8b) Over the past month, how much muscle cramping have you had?**	None	Mild	Moderate	Severe
**8c) What impact have muscle cramps had on your ability to carry out daily activities (e.g. work, household chores, childcare, social activities)?**	No impact	A small impact	A moderate impact	A large impact
**8d) Where did you experience muscle cramps? (circle all that apply)**	Hand: One Both	Arm: One Both	Foot: One Both	Leg: One Both

**Q9: Weakness**				
**9a) Over the past month how many days of the month have you had weakness?**	0 days (continue to question 10)	1 to 5 days	6 to 15 days	16 to 30 days
**9b) Over the past month, how much weakness have you had?**	None	Mild	Moderate	Severe
9c) What impact has weakness had on your ability to carry out daily activities (e.g. work, household chores, childcare, social activities)?	No impact	A small impact	A moderate impact	A large impact
**9d) Where did you experience weakness? (circle all that apply)**	Hand: One Both	Arm: One Both	Foot: One Both	Leg: One Both

**Q10: Fatigue**				
**10a) Over the past month how many days of the month have you had fatigue?**	0 days (continue to question 11)	1 to 5 days	6 to 15 days	16 to 30 days
**10b) Over the past month, how much fatigue have you had?**	None	Mild	Moderate	Severe
**10c) What impact has fatigue had on your ability to carry out daily activities (e.g. work, household chores, childcare, social activities)?**	No impact	A small impact	A moderate impact	A large impact

**Q11: Emotional Distress (e.g. sadness, worry, upset)**				
**11a) Over the past month how many days of the month have you had these feelings of emotional distress?**	0 days (continue to question 12)	1 to 5 days	6 to 15 days	16 to 30 days
**11b) Over the past month, how much emotional distress have you felt?**	None	Mild	Moderate	Severe
**11c) What impact have these feelings of emotional distress had on your ability to carry out daily activities (e.g. work, household chores, childcare, social activities)?**	No impact	A small impact	A moderate impact	A large impact

**Q12: Concentration difficulties**				
**12a) Over the past month how many days of the month have you had difficulties in concentrating?**	0 days (continue to question 13)	1 to 5 days	6 to 15 days	16 to 30 days
**12b) Over the past month, how much difficulty with concentrating have you had?**	None	Mild	Moderate	Severe
**12c) What impact have difficulties in concentrating had on your ability to carry out daily activities (e.g. work, household chores, childcare, social activities)?**	No impact	A small impact	A moderate impact	A large impact

**Q13: Sleep problems**				
**13a) Over the past month how many days of the month have you had problems sleeping?**	0 days (continue to question 14)	1 to 5 days	6 to 15 days	16 to 30 days
**13b) Over the past month, how much problem sleeping have you had?**	None	Mild	Moderate	Severe
**13c) What impact have sleeping difficulties had on your ability to carry out daily activities (e.g. work, household chores, childcare, social activities)?**	No impact	A small impact	A moderate impact	A large impact

Q 14: Please use the scales below to rate how much pain you had on average over the last month in each of the mentioned body areas. Please circle the number which corresponds with your level of pain with 0 being no pain and 3 being severe pain.

		None	Mild	Moderate	Severe			None	Mild	Moderate	Severe
A	**Left fingers**	0	1	2	3	I	**Right fingers**	0	1	2	3
B	**Left wrist**	0	1	2	3	J	**Right wrist**	0	1	2	3
C	**Left elbow**	0	1	2	3	K	**Right elbow**	0	1	2	3
D	**Left shoulder**	0	1	2	3	L	**Right shoulder**	0	1	2	3
E	**Left hip**	0	1	2	3	M	**Right hip**	0	1	2	3
F	**Left knee**	0	1	2	3	N	**Right knee**	0	1	2	3
G	**Left ankle**	0	1	2	3	O	**Right ankle**	0	1	2	3
H	**Left toes**	0	1	2	3	P	**Right toes**	0	1	2	3
Q	**Neck**	0	1	2	3	R	**Back**	0	1	2	3

Q15: This question asks about how your symptoms have developed **since the time they first began.** Please select the ***one pattern of the following options that you think best describes*** how your symptoms have developed. Between the time that your symptoms first began and now, have your symptoms:

	**Please tick the *one* box which best describes your symptom pattern:**	
a) increased ***rapidly* and then remained constant** (like the line to the right):	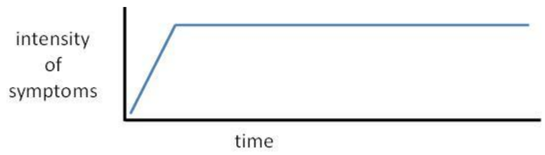	☐
b) ***gradually*** increased to their current level over time (like the line to the right):	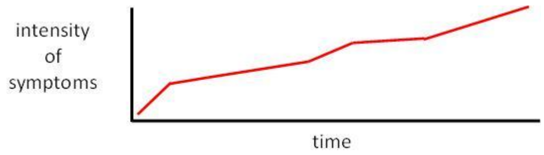	☐
c) **come and gone** increasing and decreasing though ***always with some symptoms*** (like the line to the right):	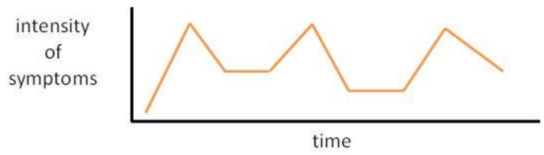	☐
d) **come and gone** with ***periods without symptoms* in between** (like the line to the right):	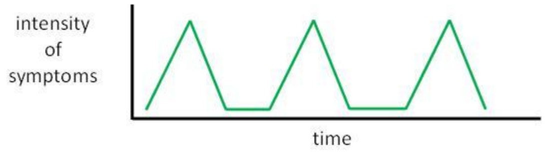	☐
e) If these do not match your symptom experience, use the space to the right to draw what your symptoms were like between the time they began and now, or describe them below:<br>________________________________<br>________________________________	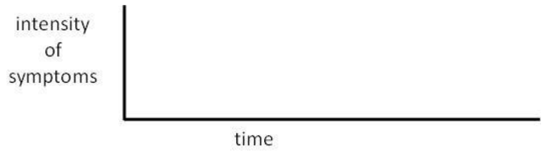	☐

Is there anything else you want to tell us about your symptoms?
Or do you want to tell us anything about the questionnaire?
Please leave your comments below:

____

____

____

____

____

____

____

____

____

____

Thank you very much!

©2014. Copyright of the SPARRA Questionnaire resides with RJ Stack (Birmingham University), K Raza (Birmingham University), LH van Tuyl (VUmc) and D van Schaardenburg (Reade). Version 1, only for use in collaboration with the authors. Validation of the SPARRA Questionnaire is supported by EULAR.
