# Supplementary material for: Symptoms in first-degree relatives of patients with rheumatoid arthritis: evaluation of cross-sectional data from the symptoms in persons at risk of rheumatoid arthritis (SPARRA) questionnaire in the PRe-clinical EValuation of Novel Targets in RA (PREVeNT-RA) Cohort
Source: Arthritis Res Ther. 2021 Aug 11;23:210. doi: 10.1186/s13075-021-02593-w (PMC8356426; doi:10.1186/s13075-021-02593-w)
Supplement: Supplementary file 2 — Additional file 2: Table 1 Specific details of seropositivty. Table 2 Characteristics and associations with symmetrical and small joint pain in females only. Table 3: Symptoms and associations with symmetrical and small joint pain in females only (N=631) [file 13075_2021_2593_MOESM2_ESM.docx]

Additional file 2

Table 1: Specific details of seropositivty

|  | RF negative | RF positive |
| --- | --- | --- |
| ACPA negative | 827 (95.1%) | 30 (1.0%) |
| ACPA positive | 9 (3.4%) | 4 (<1%) |

Table 2: Characteristics and associations with symmetrical and small joint pain in females only.

| Characteristics |  | Number (%) without symmetrical and small joint pain, unless otherwise specified | Number (%) with small and symmetrical joint pain, unless otherwise specified | Univariate logistic regression Odds Ratio (95% CI)^$^ | Multivariate logistic regression Odds Ratio (95% confidence interval) ^$^ |
| --- | --- | --- | --- | --- | --- |
| Age (continuous) (Mean (SD)) | | 50.1 (11.5) | 56.3 (11.1) | 1.05 (1.03, 1.07) | 1.03 (1.01, 1.06) |
| Smoking status | Never smoker | 317 (90.0) | 35 (9.9) | Reference | Reference |
|  | Former smoker | 170 (85.0) | 30 (15.0) | 1.60 (0.95, 2.69) | 1.61 (0.89, 2.91) |
|  | Current smoker | 27 (81.8) | 6 (18.2) | 2.01 (0.78, 5.21) | 1.78 (0.60, 5.29) |
| Average units of alcohol consumed per week (continuous) |  | 6.2 (7.0) | 6.1 (7.1) | 1 (0.97, 1.04) | 1 (0.96, 1.04) |
| BMI category | Underweight | 12 (100) | 0 | Null | Null |
|  | Normal weight | 253 (92.3) | 21 (7.7) | Reference | Reference |
|  | Overweight | 152 (83.5) | 30 (16.5) | 2.38 (1.31, 4.30) | 1.80 (0.95, 3.42) |
|  | Obese | 97 (82.9) | 20 (17.1) | 2.48 (1.29, 4.78) | 1.86 (0.84, 4.13) |
| Educational left education | Less than 16 years | 27 (73.0) | 10 (27.0) | 2.08 (0.92, 4.69) | 0.84 (0.32, 2.22) |
|  | 16-17 years | 191 (84.9) | 34 (15.1) | Reference | Reference |
|  | 18-20 years | 146 (92.4) | 12 (7.6) | 0.46 (0.23, 0.92) | 0.56 (0.27, 1.17) |
|  | 21+ years | 150 (90.9) | 15 (9.1) | 0.56 (0.30, 1.07) | 0.75 (0.37, 1.52) |
| IMD quintile | 1 | 36 (80.0) | 9 (20.0) | Reference | Reference |
|  | 2 | 84 (90.3) | 9 (9.7) | 0.43 (0.16, 1.17) | 0.48 (0.16, 1.49) |
|  | 3 | 115 (88.5) | 15 (11.5) | 0.52 (0.21, 1.29) | 0.60 (0.21, 1.69) |
|  | 4 | 134 (87.0) | 20 (13.0) | 0.60 (0.25, 1.42) | 0.76 (0.27, 2.12) |
|  | 5 | 145 (89.0) | 18 (11.0) | 0.50 (0.21, 1.20) | 0.47 (0.16, 1.33) |
| Depression | No | 432 (90.8) | 44 (9.2) | Reference | Reference |
|  | Yes | 82 (75.2) | 27 (24.8) | 3.23 (1.89, 5.52) | 2.92 (1.58, 5.39) |
| CRP | Normal | 443 (88.8) | 56 (11.2) | Reference | Reference |
|  | Elevated | 71 (82.6) | 15 (17.4) | 1.67 (0.90, 3.11) | 1.15 (0.55, 2.41) |
| Antibody status | Seronegative | 490 (88.1) | 66 (11.9) | Reference | Reference |
|  | Seropositive | 24 (82.8) | 5 (17.2) | 1.55 (0.57, 4.19) | 2.1 (0.71, 6.19) |
| Pregnancy ever | No | 98 (94.2) | 6 (5.8) | Reference | Reference |
|  | Yes | 416 (86.5) | 65 (13.5) | 2.55 (1.07, 6.06) | 2.05 (0.76, 5.54) |
| Number breastfed | 0 | 228 (87.7) | 32 (12.3) | Reference | Reference |
|  | 1 | 89 (89.9) | 10 (10.1) | 0.80 (0.38, 1.70) | 0.71 (0.31, 1.66) |
|  | 2 | 134 (85.9) | 22 (14.1) | 1.17 (0.65, 2.10) | 1.07 (0.53, 2.16) |
|  | 3 | 63 (90.0) | 7 (10.0) | 0.79 (0.33, 1.88) | 0.54 (0.2, 1.42) |
| Ever used hormonal contraception | No | 86 (86.0) | 14 (14.0) | Reference | Reference |
|  | Yes | 428 (88.3) | 57 (11.8) | 0.82 (0.44, 1.53) | 0.84 (0.41, 1.74) |
| Ever used HRT | No | 442 (90.6) | 46 (9.4) | Reference | Reference |
|  | Yes | 72 (74.2) | 25 (25.8) | 3.34 (1.93, 5.77) | 2.21 (1.15, 4.23) |

^$^ Difference in proportions is significant if different to one.

Table 3: Symptoms and associations with symmetrical and small joint pain in females only (N=631)

| Symptom present | Number without symmetrical and small joint pain (%) | Number with small and symmetrical joint pain (%) | Univariate logistic regression Odds Ratio (95% CI)^$^ | Multivariate logistic regression Odds Ratio (95% confidence interval) ^$^ |
| --- | --- | --- | --- | --- |
| joint pain | 37 (7.1) | 42 (37.2) | 7.69 (4.63, 12.77) | 2.55 (1.24, 5.24) |
| joint swell | 65 (10.9) | 14 (40.0) | 5.45 (2.64, 11.23) | 0.74 (0.25, 2.24) |
| joint stiff | 44 (7.9) | 35 (46.1) | 9.91 (5.74, 17.12) | 2.55 (1.15, 5.63) |
| joint burn | 67 (11.0) | 12 (54.6) | 9.7 (4.04, 23.3) | 4.42 (1.38, 14.13) |
| joint tingling | 64 (10.5) | 15 (79.0) | 32.1 (10.3, 99.7) | 13.36 (3.58, 49.80) |
| Colour | 76 (12.2) | 3 (37.5) | 4.32 (1.01, 18.43) | 0.72 (0.10, 5.14) |
| Cramp | 62 (11.0) | 17 (24.6) | 2.64 (1.44, 4.84) | 0.96 (0.41, 2.22) |
| Weakness | 56 (9.8) | 23 (40.4) | 6.26 (3.45, 11.36) | 1.41 (0.61, 3.27) |
| Fatigue | 40 (7.7) | 39 (34.2) | 6.20 (3.74, 10.26) | 2.61 (1.27, 5.35) |
| Distress | 51 (9.8) | 28 (25.0) | 3.06 (1.83, 5.13) | 1.04 (0.48, 2.23) |
| concentration difficulties | 60 (10.3) | 19 (41.3) | 6.16 (3.23, 11.73) | 0. 91 (0.33, 2.50) |
| Sleep disturbances | 46 (9.3) | 33 (24.4) | 3.16 (1.93, 5.20) | 0.95 (0.46, 1.96) |

^$^ Difference in proportions is significant if different to one.
